# Characterization and Testing the Efficiency of *Acinetobacter baumannii* Phage *vB-GEC_Ab-M-G7* as an Antibacterial Agent

**DOI:** 10.3389/fmicb.2016.01590

**Published:** 2016-10-04

**Authors:** Ia Kusradze, Natia Karumidze, Sophio Rigvava, Teona Dvalidze, Malkhaz Katsitadze, Irakli Amiranashvili, Marina Goderdzishvili

**Affiliations:** ^1^G. Eliava Institute of Bacteriophages, Microbiology and VirologyTbilisi, Georgia; ^2^Ivane Javakhishvili Tbilisi State UniversityTbilisi, Georgia; ^3^Institute of Medical Research, Ilia State UniversityTbilisi, Georgia

**Keywords:** *Acinetobacter baumannii*, bacteriophage, phage therapy, animal model, wound infection

## Abstract

*Acinetobacter baumannii* is a gram-negative, non-motile bacterium that, due to its multidrug resistance, has become a major nosocomial pathogen. The increasing number of multidrug resistant (MDR) strains has renewed interest in phage therapy. The aim of our study was to assess the effectiveness of phage administration in *Acinetobacter baumannii* wound infections in an animal model to demonstrate phage therapy as non-toxic, safe and alternative antibacterial remedy. Using classical methods for the study of bacteriophage properties, we characterized phage *vB-GEC_Ab-M-G7* as a dsDNA myovirus with a 90 kb genome size. Important characteristics of *vB-GEC_Ab-M-G7*include a short latent period and large burst size, wide host range, resistance to chloroform and thermal and pH stability. In a rat wound model, phage application effectively decreased the number of bacteria isolated from the wounds of successfully treated animals. This study highlights the effectiveness of the phage therapy and provides further insight into treating infections caused by MDR strains using phage administration.

## Introduction

*Acinetobacter baumannii* is a gram-negative, non-motile bacterium that has become a major nosocomial pathogen due to its multidrug resistance. *A. baumanni* strains have been isolated which are resistant to almost all antibiotics, including a high prevalence of resistance to carbapenems which has been reported worldwide since the 1990’s (Kempf and Rolain, 2012; Ahmed et al., 2016; Nowak and Paluchowska, 2016). Most of the strains are still sensitive *in vitro* to colistin, an antibiotic which was considered toxic for a long time (Montero et al., 2004). Recent studies suggest it can actually be used as an efficient antimicrobial agent (Michalopoulos and Falagas, 2011). But colistin resistant *A. baumannii* strains have already been reported (Lopez-Rojas et al., 2011; Kempf and Rolain, 2012; Gupta et al., 2016; Yilmaz et al., 2016). Currently there is no remedy to effectively remove *A. baumannii* from the hospital environment, as this microbe is resistant to dehydration, chemical sanitizers and detergents (Yang et al., 2010). As a result, the risk of *A. baumannii* nosocomial infection is increasing.

The worldwide spread of MDR strains of a number of different pathogens has renewed interest in bacteriophage therapy. Bacteriophages are viruses which infect and lyse bacteria. Lytic phages for the treatment of infections were first introduced by Felix d’Herelle in 1920 (Matsuzaki et al., 2005). Due to their observed efficacy and recognized specificity, phages have been used as a treatment modality in the Formers Soviet Union and Eastern Europe, especially in Georgia, for decades; much successful work was also carried out in France and elsewhere (Matsuzaki et al., 2005; Sulakvelidze, 2005; Kutter et al., 2010).

The study of phage therapy in animal models is an essential bridge between *in vitro* and clinical studies. Mice with artificial burns, pneumonia, pulmonary infections with various microbes (*Pseudomonas* spp., *Staphylococcuss* spp., *Enterococcuss* spp., *Klebsiella pneumoniae, Escherichia coli*) were successfully treated with phages (Biswas et al., 2002; Chibani-Chennoufi et al., 2004; Vinodkumar et al., 2005; McVay et al., 2007; Debarbieux et al., 2010; Morello et al., 2011). Although several *A. baumannii* phages have been isolated and characterized in terms of potential therapeutic application (Soothill, 1992; Yang et al., 2010; Popova et al., 2012), a very few *in vivo* trials for *A. baumannii* phages have been reported: Wang et al. (2016), showed successfully use of phage intranasal application for treatment pneumonia on mice model. All mice survived after intranasal application of phages published by Jeon et al. (2016) as well. Uncontrolled diabetic rats were used for infected wound modeling for study phage therapy effectiveness by Shivaswamy et al. (2015), where ones more was demonstrated *A. baumannii* phage prospects for treatment of MDR bacteria caused infections. Several studies done in Georgia at Eliava Institute of BMV highlight phage therapy advantages and effectiveness, as well (Kutateladze and Adamia, 2008, 2010). Furthermore, a range of phages targeting the organism in question are required to successfully develop a phage therapy approach. In our study we have characterized a promising new lytic *A. baumannii* bacteriophage *vB_Ab-M-G7* and report its potential in phage therapy on a rat wound model, using both its original and a French clinical strain of *A. baumannii*.

## Materials and Methods

### Bacterial Strains, Identification

Clinical isolates of *Acinetobacter baumannii G7* and *T-10* were used in this study. *A. baumannii* strains *G7* –isolated in Georgia from an injured soldier during the Georgian–Russian War in 2008 as previously described (Kusradze et al., 2011), was used for isolation and concentration of the reported phage. Strain *T-10* was isolated from a patient in the hospital de la Timone, Marseille, France. Bacteria were grown at 37°C in Brain Heart Infusion broth and agar, and in Herellea Agar (Jawad et al., 1994). Matrix-assisted Laser Desorption Time-of-Flight Mass Spectrometry (MALDI-TOF MS) (Autoflex, Bruker Daltonics) with the flex control software (Bruker Daltonics) was used for identification these strains. A score value >1.9 is considered adequate for identification at the species level (Seng et al., 2009).

### Phage Isolation, Concentration, Purification

Bacteriophage was isolated from sewage water. After overnight incubation of the sewage samples with microbial culture in Brain Heart Infusion Broth (BHIB) at 37°C, samples were centrifuged at 5000 *g* for 20 min and filtrated and the supernatant was checked for phages by a standard spot test: overnight host strain (18–24 h.) diluted in BHIB 10^8^ cfu ml^-1^ were streaked onto a Petri plate with 1.5% agar. After drying of the lines (15–20 min), 0.05 ml of each supernatant was spotted onto each line. Plates were incubated at 37°C for 18 h. After incubation, the appearance of lysis zones on the bacterial lines shows the presence of phages (Garbe et al., 2010; Karumidze et al., 2013). Phage concentration and plaque morphology was determined by serial dilution of the phage lysate, 1 ml serial diluted phage and 0, 1 ml indicator bacteria was added to 4.5 ml 46°C 0.7% soft-agar and poured onto the 1, 5% bottom agar on Petri dishes. After 30 min the plate was incubated at 37°C. The results were counted after 18–24 h. Plaque Purification of bacteriophage was repeated 15–20 times. Purified phage was amplified and titer in the filtrate was assessed by using the double-agar layer method (Lin et al., 2010). The phage lysate was stored at 4°C.

### Phage Host Range Spectrum, Single Step Growth Curve, Adsorption Rate

The phage host range spectrum was determined on 200 *A. baumannii* strains (Eliava Collection) using a standard spot test protocol (Garbe et al., 2010; Karumidze et al., 2013). To determine phage growth characteristics (latent period, burst size), experiments were carried out according to the previously described (Drulis-Kawa et al., 2011; Rigvava et al., 2013), with some modifications. *A. baumannii* strain was grown in 10 ml BHIB until the exponential growth phase, 0, 1 ml phage with titer 10^8^ pfu ml^-1^ was added at a multiplicity of infection (MOI) of 0.1. Samples were taken periodically for the determination of phage growth parameters.

To calculate the phage adsorption rate, 1 ml bacterial suspension (10^7^ cfu ml^-1^) and 1 ml phage lysate (10^8^ pfu ml^-1^) were mixed and incubated at 37°C. 0.1 ml samples were taken at 0’, 5’, 7’, 10’, 15’, and 20’ min and added to 9,9 ml BHIB and 0,4 ml chloroform. Samples were mixed well and plated using the double agar-layer method to determine the amount of unabsorbed phages.

### pH, Chloroform and Thermal Stability Tests

The phage preparation (1 × 10^10^ pfu ml^-1^) was incubated at 37, 50, and 70°C for 24 h. From chloroform stability tests, 1 ml (1 × 10^10^ pfu ml^-1^) bacteriophage was mixed with 0.4 ml chloroform and incubated for 24 h at room temperature. For pH stability studies, phage at 1 × 10^10^ pfu ml^-1^ was incubated at pH 3, 5, 7, 9, and 11 for 24 h. For all three experiments samples were taken at 5 and 24 h and the phage titer was determined using the double-agar-layer method. BHI broth and BHI agar were used.

### Electron Microscopy

Phage morphology was determined by transmission electron microscopy as following: a 5 μl sample was placed onto a fresh glow discharged pioloform coated grid; the excess sample was removed and the grid was washed using 2 × DDW (Double Distilled Water); 2 drops of 1% uranyl acetate were added, the excess was immediately removed and the grid was allowed to air dry. Samples were viewed on a JEOL JEM 1400 TEM with an accelerating voltage of 80 kV. Images were captured using a Megaview III digital camera with iTEM software.

### Phage DNA Isolation, Restriction Endonuclease Analysis, PFGE

Before phage DNA isolation, the phage lysate was treated with DNAse and RNAse to remove residual bacterial debris. Standard PCR (Polymerase chain reaction) was used to verify the purity of the phage lysate with universal primers (536F CAGCAGCCGCGGTAATAC, Rp2 ACGGCTACCTTGTTACGACTT) targeting the 16S rRNA gene as previously described (Bittar et al., 2008). Phage DNA isolation was performed by using High Pure Viral Nucleic Acid Qiagen Kit (QIAGEN, Courtaboeuf, France) according to the manufacturer’s instructions. For digestion of phage DNA 8 different restriction endonucleases (BamHI, EcoR I, EcoRV, HindIII, HincII, PstI, DpnI, SpeI) were used according to the instructions provided by manufacturer. For separation of the DNA fragments, electrophoresis was done using 0.8% agarose gel. Restriction digestions were repeated three times. Pulsed-field gel electrophoresis was performed as described in Lin et al. (2010) for determination of the phage DNA size.

### Preparation for Animal Wound Model

Animal experiments were done according to the Animal Rights Committee in Georgia, which fully recognizes the Universal Declaration of Animal Rights.

A total of 30 adult rats weighing 200–300 g were used in this study. Experimental wounds were done as following: each rat was anesthetized and secured to the operating table. After being shaved, the skin was cleaned in aseptic conditions with a 5% iodine solution and a dorsal full-thickness 1.5 × 1.5cm surgical wound was administered. Interrupted stitches were used to secure a plastic cover to avoid contamination, as well as for procedures which were performed on the animals daily.

*Acinetobacter baumannii T-10* and *G7* were used for infection and phage *vB-GEC_Ab-M-G7*was applied as a therapeutic remedy. *A. baumannii G7* was the original host for phage *vB-GEC_Ab-M-G7*, titer on this strain was 1 × 10^10^ pfu ml^-1^. *A. baumannii T-10* was chosen randomly from the strains sensitive to phage *vB-GEC_Ab-M-G7*, phage titer on this strain was 2 × 10^8^ pfu ml^-1^. Phage was diluted to receive 5 × 10^7^ pfu ml^-1^.

### Experimental Animal Wound Model

The 30 rats with experimental wounds were divided randomly into six groups, each containing five rats. Group I was aseptic wound modeling – no bacteria were added, no phage. Group II tested phage therapy of the aseptic wound (therapeutic dose) with 1ml phage application (5 × 10^7^ pfu ml^-1^), no bacteria were added. Group III – infected wound modeling, 1ml of *Acinetobacter baumannii T-10* 5 × 10^8^ cfu ml^-1^ was applied, no phage were added. Group IV tested phage therapy of the wound infected with 1 ml of *A. baumannii T-10* (5 × 10^8^ cfu ml^-1^), 1ml of phage (5 × 10^7^ pfu ml^-1^) were applied 12 h after infection. Group V – infected wound modeling, infected with 1 ml of *Acinetobacter baumannii G7* (5 × 10^8^ cfu ml^-1^), no phage were added. Group VI -phage therapy of the infected wound (1 ml of *A. baumannii G7* 5 × 10^8^ cfu ml^-1^) with 1ml phage (5 × 10^7^ pfu ml^-1^) applied after 12 h from infection. Additional, phage *vB-GEC_Ab-M-G7* (for groups II, IV, and VI) were added on the wounds every 24 h for 6 days. Samples were taken before phage application at time points 0’ (12’), 24’, 48’, 72’, and 144’ hours using a sterile swab. 4.5 ml of BHI broth was added to the swab tube and was first titered for bacterial count and then was filtered (0.22 μm) and titered for phage, according to previously stated methods. The bacterial titer was determined using Herellea agar, to avoid contamination. At the end of the experiment, every animal was euthanized in accordance with the law of animal rights.

## Results

### Bacterial Strains

Microbial strains *T-10* and *G7* were identified as *A. baumannii* using MALDI-TOF MS, with score of 2.224 and 2.272 respectively.

### Phage Properties

**FIGURE 1:**
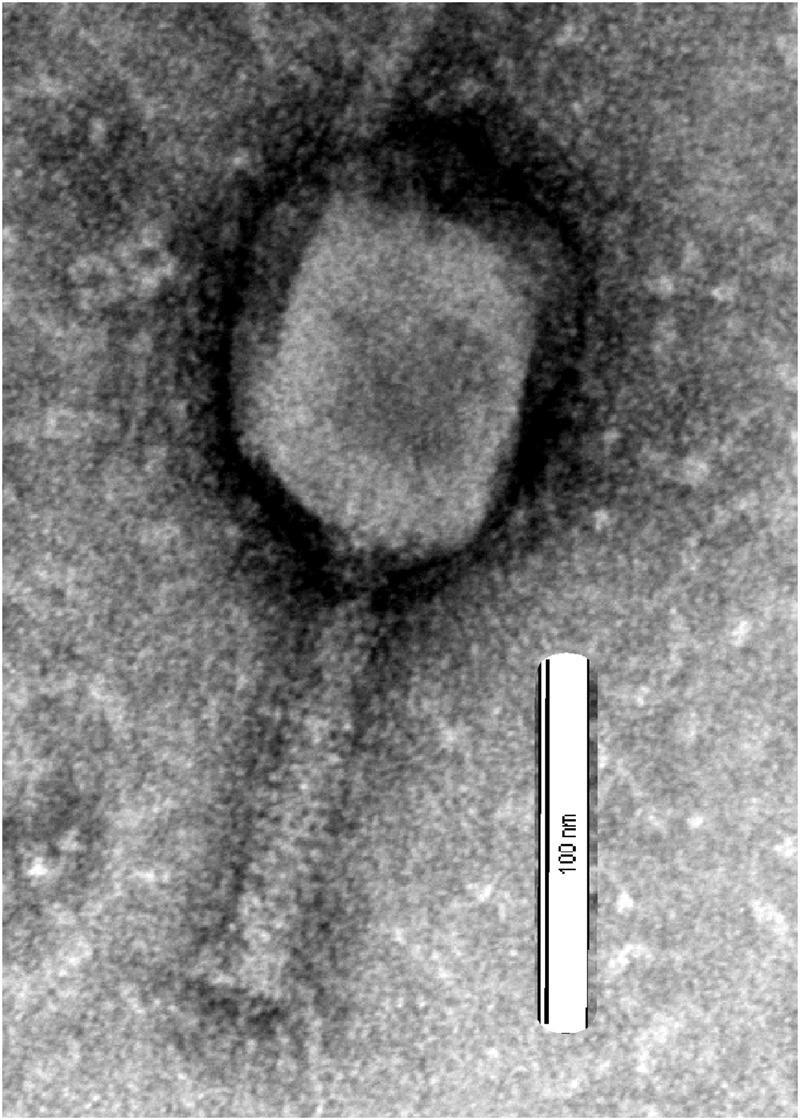
**Electron micrographs of phage *vB_Ab-M-G7*.** Bar corresponds to 100 nm.

Phage *vB-GEC_Ab-M-G7*was isolated from sewage using *A. baumannii G7* as the host. Using phage purification methods, concentration and titration, a pure, high titer (10^10^ pfu ml^-1^) stock of *A. baumannii* phage was obtained, which had small plaque morphology on Petri dishes using the double-agar-layer method. The study of phage morphology by transmission electron microscopy showed that the phage has an icosahedral head, about 100 nm in diameter and a 120 nm long contractile tail, thus belongs to Myoviridae (Matsuzaki et al., 2005) (**Figure 1**). It was named *vB-GEC_Ab-M-G7* (phage *phi G7*) according to the scheme for the nomenclature of viruses of bacteria (Kropinski et al., 2009).

**FIGURE 2:**
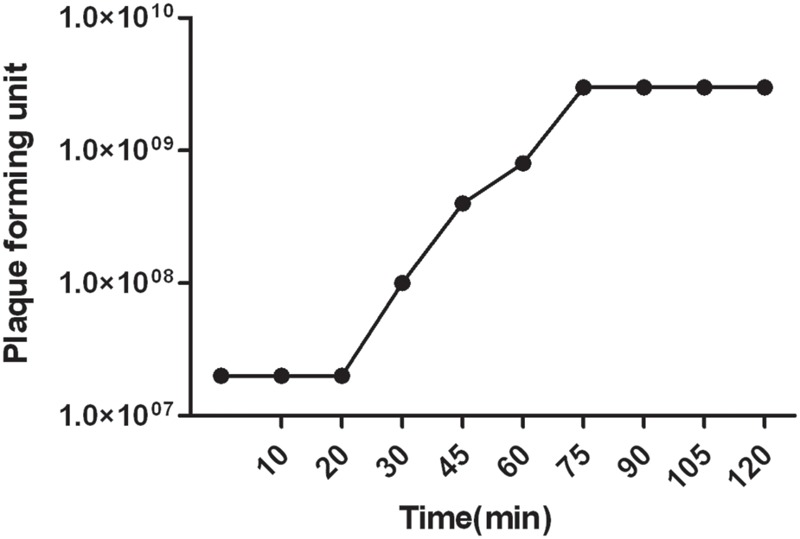
**Single step growth curve of phage *vB_Ab-M-G7*.** The latent phase takes average 20 min and the phage produces about 120 pfu ml^-1^ per infected cell.

Host range spectrum studied on 200 *A. baumannii* strains showed that phage *vB-GEC_Ab-M-G7*was able to infect 68% of the *A. baumannii* strains. The latent period of phage *phi G7* was 20 min and the burst size was 120 pfu per infected cell. Most phages (91.1%) were adsorbed within 7 min (**Figure 2**).

**FIGURE 3:**
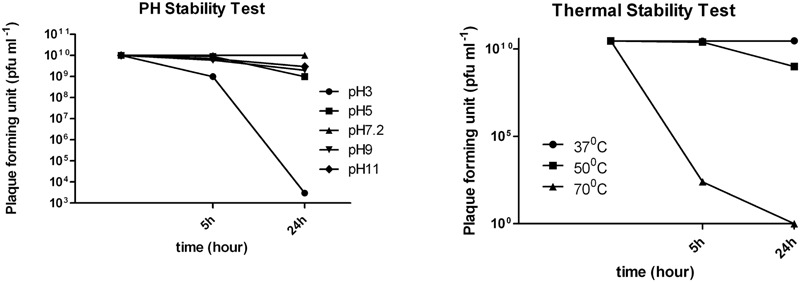
**pH and thermal stability of phage *vB_Ab-M-G7***.

Thermal stability experiments showed that phage retained 100% activity after incubation at 37°C and almost 90% of phages were viable after a 24 h incubation at 50°C (**Figure 3**). However, after 24 h at 70°C no active phages were found. Phage *phi G7* was stable after 24 h of chloroform treatment and over a pH range of 3–11 for 5 h; by 24 h the phage titer was reduced to 10^3^ pfu ml^-1^ at pH 3, but it remained unaffected within the pH range 5–11(**Figure 3**).

PFGE showed that the phage DNA size was 90 kb. None of the 8 (BamHI, EcoR I, EcoRV, HindIII, HincII, PstI, DpnI, SpeI) restriction endonucleases used in this study digested phage vB_Ab-M-G7, although they did digest the DNA of other phages.

### Phage Therapy in Infected Animal Wound Model

All of the Group I animals survived and no *A. baumannii* strains were isolated (grown in Herellae agar) from the samples taken from them, indicating that the wound modeling was done in sterile conditions and this artificial wound did not affect the rats’ mortality.

To examine the toxicity of phage, the wounded but not infected experimental Group II was used, where only phage *vB-GEC_Ab-M-G7*was applied to the artificial wound. All of the wounded rats survived, indicating that phage *phi G7* was not toxic. Samples taken from the animals showed no contamination with *A. baumannii* and the titer of the phage was not more than 2 × 10^1^± 1.7(SD) pfu ml^-1^ in each samples taken during the experiments (**Table 1**).

**Table 1:** Alternation of the bacterial titer in phage treated infected wounds.

Groups	Rats (200–300 g)	Bacterial Infection (1 ml 5 × 10^8^ CFU” ml^-1^)	Delay Before Phage Application	Phage Application 1 ml 5 × 10^7^ PFU ml^-1^ (6 days)	Results
I	Aseptic wound	–	–	–	No strain was isolated
II	Aseptic wound	–	12 h	*Phi G7*	No strain was isolated; 2 × 10^1^ ± 1.7(SD)’ PFU”’ ml^-1^
III	Infected wound	*A. baumannii T10*	–	*–*	8 × 10^6^ ± 0.9(SD) CFU ml^-1^ increased to 9 × 10^7^± 1.6(SD) CFU ml^-1^
IV	Infected wound	*A. baumannii T10*	12 h	*Phi G7*	7 × 10^6^± 0.9(SD) CFU ml^-1^ decreased to 9 × 10^2^± 1.3(SD) CFU ml^-1^
V	Infected wound	*A. baumannii G7*	–	*–*	1 × 10^7^ ± 1.8(SD) CFU ml^-1^ increased to 2,5 × 10^8^ ± 2.9(SD) CFU ml^-1^
VI	Infected wound	*A. baumannii G7*	12 h	*Phi G7*	1,4x10^7^± 0.9(SD) CFU ml^-1^ decreased to 3 × 10^2^± 1.9(SD) CFU ml^-1^

*’, Standard Deviation; ”, Colony Forming Unit; ”’, Plaque Forming Unit.*

**FIGURE 4:**
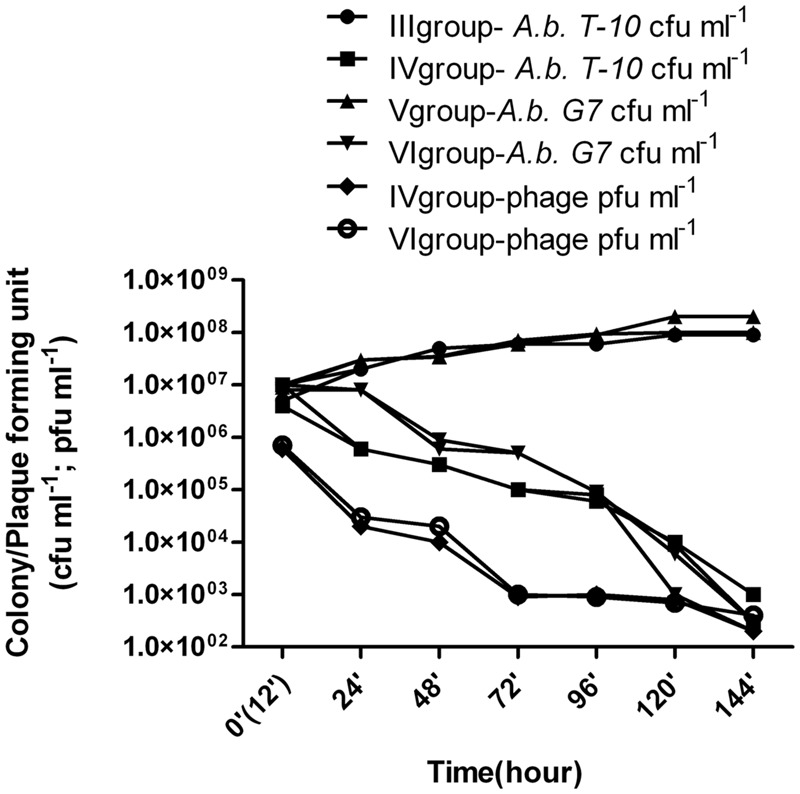
**Phage therapy results in animal wound model.** *III group* – *A. baumannii T-10* strain titer was increased from ≈8 × 10^6^± 0.9 (standard deviation – SD) cfu ml^-1^ to ≈ 9 - 10^7^± 1.6(SD) cfu ml^-1^; *IV group*- alternation of the bacterial and phage titer during the 7 days were as following, ≈7 × 10^6^± 0.9(SD)cfu ml^-1^ to ≈ 9 × 10^2^± 1.3(SD) cfu ml^-1^; ≈6 × 10^5^± 1.5(SD) pfu ml^-1^ to ≈ 3 × 10^2^± 1.6(SD) pfu ml^-1^, respectively; *V group* – *A. baumannii G7* strain titer was increased from ≈1 × 10^7^± 1.8(SD) cfu ml^-1^ to ≈ 2.5 × 10^8^± 2.9(SD) cfu ml^-1^; *VI group*- alternation of the bacterial and phage titer during the 7 days were as following, ≈1.4 × 107± 0.9(SD) cfu ml^-1^ to ≈3 × 10^2^± 1.9(SD) cfu ml^-1^; ≈7 × 10^5^± 1.5(SD) pfu ml^-1^ to ≈2 × 10^2^± 1.9(SD) pfu ml^-1^, respectively. Phage and bacteria titer is given in arithmetical average.

In groups III and V, the animals were administered 1 ml of *A. baumannii T-10* or *G7* (5 × 10^8^ cfu ml^-1^). Purulent and inflammatory processes after the second day of infection could be easily observed and became heavier at the end of experiment, indicating of infection and not colonization. Wounds infected with *A. baumannii G7* were observed to be more serious and complicated, and in group V the rate of mortality was 30%, by the fifth day of the experiment. Microbial growth is shown in **Figure 4** and **Table 1**. Samples were tested for phage contamination; no phage plaques were detected in the plated samples from animal groups III and V.

Group VI – 12 h after infection with 1ml of *A. baumannii G7* (5 × 10^8^ cfu ml^-1^), 1 ml of 5 × 10^7^ pfu ml^-1^ phage *phi G7* was applied to the wound during 6 days. During treatment the rats were active and some of them were aggressive. Before the phage application no inflammatory processes were present. By the third, day purulent processes appeared which fully vanished by the end of the experiment. The reduction of the infection symptoms was correlated with decreasing of the bacteria titer to an average 3 × 10^2^± 1.9(SD) cfu ml^-1^ and the phage titer dropped from 7 × 10^5^± 1.5(SD) 2 × 10^2^± 1.9(SD) pfu ml^-1^ (**Figure 4**, **Table 1**). All the animals survived.

Group IV -*A. baumannii T-10* showed less virulence than *A*. *baumannii G7*, (groups III and V). Slight inflammations, as well as a little purulence were characteristics of the treatment processes, but it fully disappeared before days 5 and 6. Decreasing of the bacterial and phage titer, respectively, are given in **Figure 4** and **Table 1**.

## Discussion

Due to the increase in the number of antibiotic resistant microbes, phage therapy is considered as alternative treatment for MDR bacterial infections (Chibani-Chennoufi et al., 2004; Sulakvelidze, 2005; Debarbieux et al., 2010; Kutter et al., 2010). Phage preparations are widely used to treat infections caused by *E. coli, Pseudomonas aeruginosa, Salmonella* spp., *Enterococcus faecium, Streptococcus* spp., *Staphylococcus aureus* and *Proteus* spp. in countries of the former Soviet Union (Matsuzaki et al., 2005; Sulakvelidze, 2005), but *A. baumannii* phages have not yet been used as therapeutic tools. In our study we have shown effectiveness of the phage *vB-GEC_Ab-M-G7in vivo*, correlated with its high *in vitro* activity.

Phage *phi G7* is a tailed virus with 90kb double stranded DNA genomes, belonging to the *Myoviridae* family. Some characteristics of phage *phi G7* are: a short latent period and large burst size, quite wide host range (68% on 200 clinical strains), resistant to chloroform and stabile in different thermal and pH ranges. Resistance to different restriction enzymes presumably helps make this phage active over such a wide spectrum; sequencing of the phage will determine whether this involves selection against all such restriction sites, as was observed for staph phage SB-1 (Kvachadze et al., 2011) or some other mechanism, such as the substitution of an unusual base seen in coliphage T4 and its relatives (Miller et al., 2003). All these characteristics help made phage *phi G7* a very promising component of a cocktail for treatment of *A. baumannii* infection. For this *in vivo* study we selected two *A. baumannii* strains: *G7* and *T-10* that were correctly identified as *A. baumannii* using MALDI-TOF MS. Patient-delivered *A. baumannii G7* is the host for phage *phi G7* and strain *T-10* was chosen randomly from *phi G7* sensitive strains for this experiment. No phage *phi G7* adaptation procedures were carried out using the *A. baumannii T-10* strain. We wanted to show the effectiveness of the phage *phi G7* therapy in an artificial infected rats wound model and at the same time to investigate potential differences in the therapeutic effectiveness of this phage, for treatment of infections caused by both host and randomly selected *A. baumannii* strains.

We have demonstrated in this animal wound model that the phage application did not have a toxic effect on wounded rats: in phage control group (group II) where phages were administered to the wound all the rats survived, they were feeling calm and no aggression was observed. Phage application in wound infections caused by *A. baumannii T-10* and *G7* effectively reduced the number of bacteria isolated from treated animals and all visible infection symptoms (red, swollen-purulent wound) disappeared (**Figure 4**, **Table 1**). Aggressive behavior of the infected rats fully vanished which was correlated with disappearing of infection symptoms. Rats with infections caused by the original host strain and by a randomly selected strain were treated with the same successful results. Obviously more detailed studies examining the effect of the phage dosage, timing of phage administration, pharmacokinetics will need to be undertaken before *A. baumannii* phages can be used in therapy. However, our characterization of phage *vB-GEC_Ab-M-G7* and animal experimentation illustrates its big potential for treatment of infections. We hope this study will provide further insight on treating infections caused by MDR strains using phage administration.

## Author Contributions

IK planned given research and analysed obtained results. NK, SR, and TD were involved in phage research. IA and MK performed animal experiment. MG was consulting.

## Conflict of Interest Statement

The authors declare that the research was conducted in the absence of any commercial or financial relationships that could be construed as a potential conflict of interest.
